# Supplementary material for: Facile induction of immune tolerance by an interleukin-2–TGFβ surrogate agonist
Source: Nature. 2026 Mar 11;653(8115):888–99. doi: 10.1038/s41586-026-10208-0 (PMC13190267; doi:10.1038/s41586-026-10208-0)
Supplement: Supplementary file 1 — Supplementary Fig. 1. Uncropped SDS–PAGE gels. Supplementary Table 1. Lists of signature genes used for the scRNA-seq analysis. Supplementary Table 2. DEGs induced by TGFβ and IL-2 signalling in response to IL-2–TGFβ surrogate agonist treatment during pTreg cell differentiation in vivo. Supplementary Table 3. DEGs suppressed by TGFβ and IL-2 signalling in response to IL-2–TGFβ surrogate agonist treatment during pTreg cell differentiation in vivo. [file 41586_2026_10208_MOESM1_ESM.pdf]

nature portfolio

https://doi.org/10.1038/s41586-026-10208-0

Supplementary information

Facile induction of immune tolerance by an interleukin-2–TGFβ surrogate agonist

In the format provided by the authors and unedited

Supplementary Figure 1

Extended data figure 1b

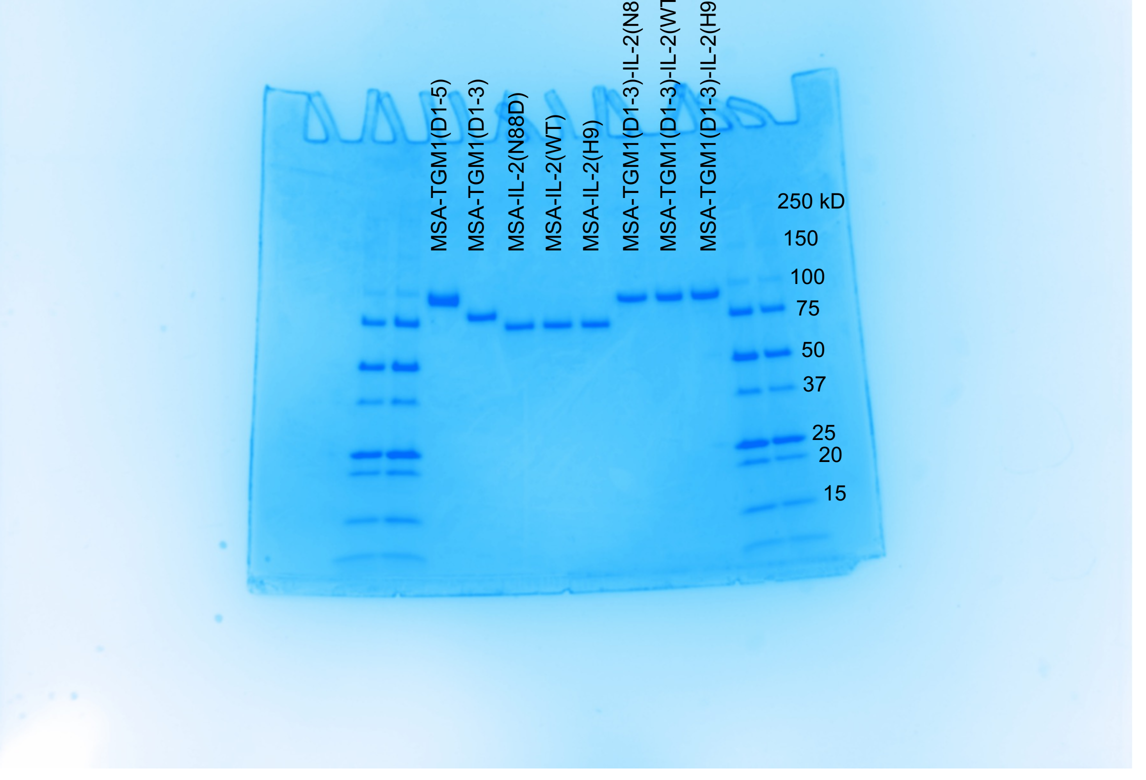

Extended data figure 9a

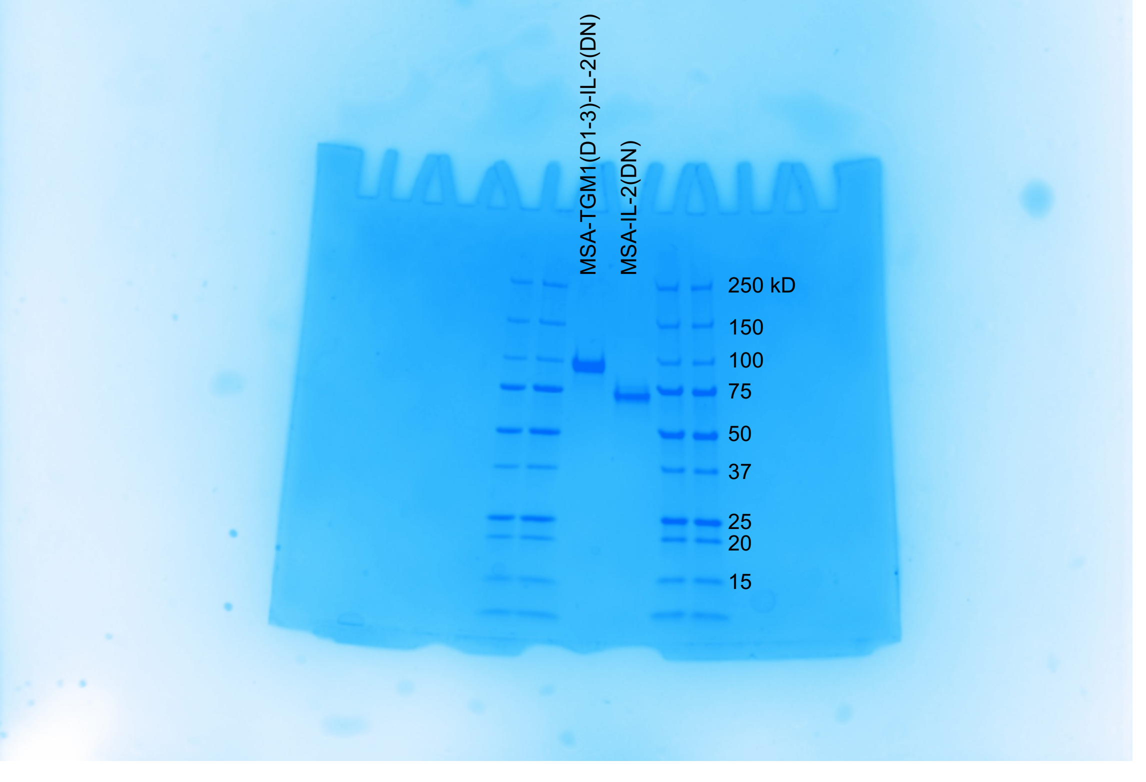

Supplementary Table 1

TGF-β-promoted signature genes

4930486L24Rik	Apol9a	Cd5l	Cxcr3	Ffar4	Gpr153	Il17f	Lhx6	Ncmap	Pipox	Rorc	Stxbp6
6030468B19Rik	Apol9b	Cd7	Cxcr4	Fmnl2	Gpr25	Il17rb	Lrrc32	Nfe2	Plau	S100a4	Syp
Abcc8	Aqp3	Cd72	Cyp11a1	Foxp3	Gpr4	Il1rn	Ly6g5b	Ngfr	Pld4	Sardh	Tbc1d16
Acsbg1	Arhgap20	Cd8a	Cyp1a1	Frmpd1	Gpr55	Itgae	Maf	Nrarp	Plekhd1	Scd1	Tgfbr1
Actr3b	Armc3	Cdh1	Dkk3	Galr3	Grb7	Jup	Mcc	Nrgn	Plscr4	Sec1	Timp2
Acvr1c	Atp1a2	Ceacam15	Dtx4	Gap43	Grifin	Kctd14	Mical2	Nt5e	Pou6f1	Sema6d	Tlr9
Acvrl1	B3galnt1	Cldn2	Dyrk4	Gcnt1	Gsta4	Kifc3	Mpp2	Ntn5	Prss23	Sfrp2	Tmeff1
Adam12	B3gnt8	Cmklr1	Emp1	Gcnt2	H2-Eb2	Klf10	Mtus1	Nucb2	Pyroxd2	Sgk1	Tmem40
Adamtsl2	Camk2n1	Col15a1	Entpd1	Gja5	Hepacam2	Klf2	Myo1e	Olfm2	Ramp1	Shisa2	Tppp
Agap1	Ccl1	Ctla2a	Etv1	Glcci1	Hrh3	Klrd1	Myo1f	Pde2a	Rasgrp2	Spatc1	Tspan7
Ahr	Ccl20	Ctla2b	Fam20a	Gm11992	Igfbp4	Lair1	Myo3b	Pdpn	Rasgrp3	Src	Upk1b
Ak7	Ccr6	Ctnnal1	Fbln2	Gm19345	Igflr1	Lcn10	Myof	Pdzd7	Rgs9	Sstr2	Vipr2
Angptl2	Cd101	Ctsw	Fbxo2	Gna14	Ikzf2	Lcn6	Nav1	Peli2	Rhoj	St6galnac3	Xcl1
Ankrd55	Cd40	Cx3cr1	Fcrl1	Gpr15	Ikzf3	Ldlrad4	Nav2	Pigz	Rnase4	Stab1	

IL-2-promoted signature genes

Dst	Abtb2	Notch2	Rragd	Castor2	Il12rb2	Gpat4	Wdr82	Ikzf4	Dio3	Socs1	Zfp608
Nabp1	Snap23	Atp1a1	Unc13b	Cux1	Mxd1	Ppp1r3b	Cish	Nacad	Klc1	Eaf2	Cndp2
Fzd7	Zscan29	Tafa3	Patj	Gpr146	Anxa4	Acsl1	6430571L13Rik	Spdl1	Arid4b	Tiam1	Aldh18a1
Atp1b1	Trib3	Mov10	Pdzk1ip1	Fbxl18	Tuba3a	Mvb12a	Amt	Adam19	Gpld1	Kcnj15	Tctn3
Cdc42bpa	Trpc4ap	Amigo1	Rnf19b	Tecpr1	Tulp3	Gfod2	Cmtm6	Kif3a	Cage1	Btbd9	Usp27x
Trp53bp2	Lama5	Gpsm2	Rcan3	Ahcyl2	Lipe	Wdr59	Myb	Gm12258	Arl10	Notch3	Foxp3
Vim	Zfp704	Elovl6	Casz1	Ube2h	Igf1r	Exoc8	Tube1	Sgsm2	Adk	D17H6S53E	Maged2
Ncs1	Phc3	Slc39a8	Cdk6	Podxl	Akap13	Mpzl2	Dip2a	Myo1d	Sh3bp5	Ppp1r10	Syne2
Acvr1c	Sfrp2	Gbp5	Nipal1	Hipk2	Rhcg	Rexo2	Txnrd1	Vat1	Oxnad1	Slc25a27	Slc4a8
Wdsub1	Adamtsl4	Gbp2	Lrrc66	Ezh2	Ticrr	Sema7a	Socs2	Metrnl	Cacnb3	Cul7	Rhoq
Gad1	Ecm1	Bach2	Rufy3	Pdia4	Swap70	Myo6	Tmtc2				

STAT5-promoted signature genes

Gpr114	Drc1	Tmprss13	Sorcs2	Il1rl1	Specc1	Camk2n1	Rcn1	Hid1	Klf2	Ppp1r3fos	Ifngr1
Myo6	Frmd5	Pdlim4	Sema4a	Nhsl2	Rap1gap2	Gpr146	Nedd4	Gpr15	Dusp3	Bmpr1a	Tanc1
ENSMUSG00000076754	Ly6c2	Ccr3	Klrg1	ENSMUSG00000074284	Gzmc	Aim1	ENSMUSG00000079138	Fasl	Prf1	Arl4c	Bcl2l11
ENSMUSG00000081264	Lama5	ENSMUSG00000097294	Enox1	Prg4	Vmn2r100	Lhfpl1	Large	Plekhg3	Ptpn4	Car5b	Dnajc15
Pigz	Stc2	Lair1	6430571L13Rik	Ptpn9	Metrnl	Ccr4	Cd7	Txndc5	Napepld	Sqle	Axin2
Upp1	Rasgrf1	Fes	Lgals7	Kcnk5	Lgals1	Fam20a	Rtn4rl1	Cdkn1a	Arnt2	Klhdc1	Nck2
Slc6a19	ENSMUSG00000076749	Magi1	Ptafr	Plat	Itgb3	Crip2	Ermn	Gna15	Cdkn2c	Vim	S1pr1
5830411N06Rik	Gbp2b	Plac8	Tmem176b	Il4	Ptgir	Anxa1	Ppm1j	Hemk1	Iqgap2	Pcyt1a	Eya2
Gzmb	Ggt1	Slpi	Ly6c1	Nckap5	F2rl2	Pde3b	Tmem64	Apol10b	Myl4	Gramd4	Rom1
Actr3b	Dapl1	Amph	Lgmn	Il9r	Pvrl1	Dlg5	Furin	A630081J09Rik	Nedd4l	Klhdc2	Pde2a
Impg1	Hpgd	Sorcs1	Enthd1	Tbc1d16	Pard3b	Cd200r4	Snai3	Runx3	ENSMUSG00000098322	Rnf144a	Rab4a
Ifitm1	Cyp11a1	1810011H11Rik	Atg9b	Plxdc1	Crip1	F2r	Mvd	Cd200r1	Gm2a	Cxcl2	Tagln2
Ifitm3	Nov	Gbp11	Tgfb3	Aven	Rhoc	Gpr35	Glipr2	Reck	Socs2	F2rl3	Ccr2
ENSMUSG00000076752	Sytl3	Atp8b4	Tmem176a	Emp1	Gpld1						

Terminal effector Treg signature genes

Il17a	Neb	Rorc	Plac8	Ppfibp1	Rab20	Ccr2	Specc1	Irf4	Gzmc	Ripply3	Kcnk7
Il1r1	Myo3b	Plekho1	Gbp8	Lgals7	Ppp1r3b	Ccr5	Trim16	Nqo2	Gzmb	Snx9	Ehd1
Ctla4	Gpr155	Mtmr11	Gbp10	Ffar2	Cpe	Ppp1r14c	Cd68	Gcnt2	Sorbs3	Agpat4	Glyat
Ccl20	Rapsn	Gbp3	Gbp6	Fxyd7	Lrrc25	Esr1	Serpinf1	Rbm24	Rcbtb2	Vmn2r100	Slc1a1
Daw1	Prr5l	Gbp2b	Grk3	Plekhf1	Dnase2a	Marcks	Tmigd1	Cap2	Lpar6	Rgs11	Gldc
Nmur1	Frmd5	Aqp3	Moxd2	Car11	Ndrg4	Gja1	Abi3	F12	Itm2b	Fam234a	Entpd1
Ackr3	Sqor	E130308A19Rik	Tmem176b	Uevld	Smco4	Trpm2	Gngt2	Ahrr	Laptm4b	Zfp523	Pik3ap1
Farp2	Adam33	Plin2	Tmem176a	Mrgpra3	Col5a3	Stab2	Nr1d1	Glrx	Matn2	Cyp4f16	Kazald1
Nckap5	Zfp937	Cdkn2c	Gpnmb	Gas2	Slc37a2	Slc17a8	Atp6v0a1	F2r	Fam83h	Zfp870	As3mt
Il10	Abhd12	Serinc2	Il12rb2	Gdpgp1	Coro2b	Socs2	Fam20a	F2rl2	Pmm1	Twsg1	Syp
Prg4	Sdcbp2	Camk2n1	Magi1	Fes	Gpx1	Irak3	1700012B07Rik	Ocln	Naga	Hbegf	Gpr34
Sccpdh	Pltp	Samd11	Mitf	Arnt2	Lrrfip2	Rhbdd3	Trib2	Kcnk5	Vdr	Snx2	Renbp
Lefty1	Mmp9	Cdk14	Lag3	Itgax	Scn11a	Plek	Lgmn	Lgals3	Acvrl1	Aldh7a1	Cysltr1
Il1rn	Spry1	Fgl2	Ptms	Itgad	Ccr9	Havcr2	Serpina3f	Rnase4	Dgkg	Alpk2	Slc25a53
Angptl2	Tmem154	Jchain	Klrb1f	Adam12	Lztfl1	Rufy1	Serpina3g	Ang	Itgb5	4930503L19Rik	Car5b
Zeb2	Sh3d19	Cxcl3	Emp1	B4galnt4	Ccr1	Fam83g	Ckb	Nynrin	Cd86	Hsbp1l1	

Colonic Rorγt^{+} Treg signature genes

Gzmb	AW112010	Maf	Arl4c	Cited4	F2rl2	Psap	Mmd	Ctsb	Cux1	Aebp2	Ndufa10
Ccr2	Tmem176b	Tmem176a	Timp2	Thy1	Txk	Abi3	Aplp2	Cdk6	Asb2	Tnfaip8	Il18r1
Itm2b	Cd47	Il10	Gm8369	Lrrc25	Nfil3	Foxo1	Capg	Gbp4	Cd4	Mical1	Zbp1
Ly6a	Rapsn	Emp1	Igtp	Pde4b	Srpk2	Gbp3	Lgmn	Gng2	Id2	Ccr1	Odc1
Lag3	Podnl1	Ccr4	Anxa2	Rnase4	Snx5	Aqp3	Inpp4b	Ptger4	Ccnd3	Crlf3	Tmsb4x
Cxcr3	S100a6	Serpina3g	Icos	Nt5e	Evi2a	Crmp1	Chchd10	Rnf138	Ech1	Ssh2	Ctla4
Mmp9	Ccr5	Socs2	F2r	Lpar6	Tesc	Npc2	Ikzf3	Hic1	Il12rb1	Ddit4	Kbtbd11
Rcbtb2	Gbp2b	S100a4	Snx2	Cyb5a	Vim	Susd3	Pitpnc1	Wnk1	Ctsd	Ctss	Cdkn2c
Ccr9	Glrx	Ptms	Ifi47	Cmtm7	Trib2	Tpst2	Cd6	Smap2	Gngt2	Txndc17	Taf6l
Lztfl1	Rorc	Gpx1	Ltb4r1	Saraf	Ehd1	Tmem64	Pyhin1	Capn2	Mthfsl	Lgals1	Trim8
Ckb											

Supplementary Table 2

DEGs induced by TGF-β and IL-2 signaling (padj < 0.0001, $log_2FC > 1$)

TGF-β alone			TGF-β + IL-2				IL-2 alone				
99		69	150		120		237			106	
Gm33799	Ldlrad4	1700025G04Rik	Vps54	Tbxa2r	Ptprj	Miat	Tnfrsf13b	Mtfr2	Stil	H2bc3	Asf1b
Gm32803	Sesn1	Treml2	Edaradd	Arhgap31	Serpinb1a	Ahr	Dctpp1	Kif11	Mt1	Ncapg2	Dscc1
Lrrc8c	Cd38	Irf5	Pik3c2a	Trpc4ap	1700012B07Rik	Dkkl1	Cdca3	Tnk2	Ccnb1	H2bc15	Knstrn
Flicr	Plekhd1	Stra6	Icos	Exoc2	Tigit	Gpr146	Cxcr3	Cdk1	Cep55	Cdc25c	Pole
Itgb7	Pmaip1	Rragd	Pacs1	Itih5	1500009L16Rik	Osbpl3	Kif14	Eya2	Runx2	Sytl3	Aunip
Eif4e3	Pros1	Ppm1l	Antxr2	Wnk1	Ltb4r1	Rapsn	Knl1	Ncaph	Car5b	Cenpn	Kif15
Fbxo27	Kcnk6	Atp6v0a1	Pgm2l1	Rbl2	Gna15	Camk2n1	Cyp2s1	Mcm10	Slc66a2	Cenph	H2ac13
F2rl1	Selplg	Gm56700	Acpp	Ighm	F2rl2	Cd55	Crybg3	Mxd3	Frmd4b	Mcm3	Hrob
Fam124b	Dkk3	Axl	Stom	Dennd1a	S100a6	Gpr160	Ube2c	Hsbp1l1	Nupr1	Rad54l	Iqgap3
Gm13481	Grb7	Grik4	Runx1	Emb	Capg	Il1r2	Tk1	Entpd1	Csrp2	Spdl1	Ociad2
Gm30292	Adam19	Bmpr2	Specc1	Gpr155	Havcr2	Ang	Dnajb13	Hip1	H2bc14	Dlgap5	Ercc6l
Golm1	Il18r1	Tmprss3	Slc25a53	F730043M19Rik	Gm57204	Morrbid	Gm12171	Nusap1	Kif22	H2ac22	Ehd4
Cldnd1	Ramp1	Nol4l	Gm36070	Arntl	Plekhf1	Il2ra	Rom1	Misp3	Mical1	Gins2	Diaph3
Cerk	Gpr34	Gata1	Gnal	Pdgfb	Slc16a6	Matn2	Lilrb4b	Nhsl2	Pif1	Pik3ap1	H1f1
Stab1	Smad3	Etfbkmt	Maf	Slc15a3	Tent5a	B4galnt4	Rnf152	Gm12169	Bub1	Trip13	Zwilch
Tnfrsf25	Gm32707	Trat1	Stk24	Arl5c	Snx9	Hacd4	Tpx2	Pbk	Clspn	H3c11	Cenpm
Cish	Fry	Rgs9	S100a13	Gm15345	Cited4	F2r	Hmgb2	Sgo2a	Gm33104	Nedd4	Bub1b
A630091E08Rik	Cyth4	Mical3	Fli1	Retreg1	Wdfy2	Nckap5	Gm40638	Plk1	Lmnb1	Ect2	Ckap2
Cd101	Tgfbr1	1600014C10Rik	Zdhhc2	Snx5	Sdc4	Ppp1r14c	Agpat4	Rnf125	Dynlt5	Top2a	Poc1a
Ncmap	Fam3c	Gm4956	D16Ertd472e	L1cam	Arl5a	Myo1f	Mxi1	H2bc11	Nckap1	H2ac11	H2bc18
Trgc1	Gm527	Socs3	Sec24a	Serinc3	A130023I24Rik	Txk	Aurkb	St3gal3	Glipr2	Melk	Chaf1a
Airn	Src	Gpr83	Sla	Spcs2	Itgae	Emp1	Cdkn1a	Ermn	Gm39792	Cip2a	Lig1
Rarg	Fut7	Myh15	Snx2	Nav2	Pvrig	Rnase4	Pmm1	Timd2	E2f8	Ccl4	Troap
Tmem176b	Or13a27	Gm20750	Perp	Gm36723	Il1r1	Crmp1	Tph1	Gm47392	Nabp1	Ticrr	H3c7
Nebl	Sit1	Icam1	Abca2	Picalm	Neb	Rorc	Lilrb4a	Dnajc15	Fbxo5	Kif20a	Ulbp1
Marcks	Ccr8	Slc17a9	Cmtm6	Sema4b	Rgs1	Furin	Cdk6	Ska1	Pclaf	Arhgap11a	Septin10
Rras2	Mast4	Cd72	Acot9	Cyba	Ccr4	Tnfrsf8	Metrnl	Rad51ap1	2210408F21Rik	Cenpi	Exo1
Itga6	Tdrkh	Rara	Il4ra	Rexo2	Lrrc25	Ppp3ca	Gm12709	AA467197	S100a10	Cks1b	Spc25
Popdc2	Il17re	Igfbp4	Podnl1	E130308A19Rik	Hbegf	Prr29	Sgk1	Atp2b4	Cd80	2700099C18Rik	Cdc20
Gtf2ird1	Foxp3	5730419F03Rik	Ticam1	Trp53i11	Niban1	Tmem163	Sytl2	Glrx	Myadm	Ska3	Rap2a
Tmem176a	Il18rap	Ston1	Rasgrp1	Cux1	Tec	Nr1d1	Rhoc	Aspm	Gm5547	Tbx21	Eldr
Tmem64	Klrb1f	She	Ubl3	Plxnd1	Gzmb	Socs2	Cenpe	Cdca2	Ccr3	Chek1	Prim1
Crybg1	Ppp1r3f	Gm56936	Lclat1	Itgb5	Tmem273	Lamc1	Plac8	Ston2	Ccna2	Cdca5	Mybl2
Cib2	Prg4	Lzts2	Slc52a3	Rbms1	Cpd	Npas2	Ckap2l	Brca1	Trim16	Paqr4	Ccne1
Slc43a2	Tle1	Sorcs2	H3c14	Gpr132	Coro2a	Il12rb1	Ctla2a	Stmn2	Nek2	Tyms	Nrm
Cry1	Stx1a	Fcrl1	Psme2b	Inpp5d	Atp6v0d2	Fam234a	Cenpf	Akap12	Clic4	Ccnf	Cenps
Glcci1	Sntb1	1110032F04Rik	Gm16146	Klhl6	Cmtm7	Ccr1	H3c3	Mt2	Pimreg	Tacc3	Aurka
Nipal1	Ikzf3	Gm49959	Armc2	Emp3	Ky	Fgl2	Alg9	Gm2a	Spc24	H2ac8	Pcyt1a
Sytl1	Foxo1	Ntn4	Tmem154	Ctnna1	Adam12	Myo3b	Lrr1	Cgas	Cdk14	Mybl1	Gins1
Dst	Pmepa1	Gm26771	Mfsd10	Nfil3	Fes	Mmp9	Foxm1	Itga2	Kif18b	Dhfr	Bard1
Krt83	Tff1	Acvr2a	Tmigd1	Phc3	Il10	Gm15270	Kif2c	Chsy1	Lair1	Hells	H2ac10
Gm28836	St6galnac3	Spsb1	Nt5e	Ccdc74a	Il23r	Naga	Gas2l3	Glis2	Bik	Fignl1	Gimap7
Tmem51	Ccr6	Ptprf	Fam83g	Tpst2	Klf12	Cysltr1	Depdc1b	Mlf1	1700017B05Rik	Sapcd2	Tcf19
Znrf3	Gm36920	H2-Ob	Actn2	Cd47	Il17a	Dennd5a	Peli2	Rrm2	Gatm	Rad51	Kif23
Macir	Col15a1	Mcoln3	Hsp90b1	Ipmk	Frmd5	Selenom	Mmp25	Aplp2	Gm5127	Cmc2	Syce2
Il7r	Dgkh	Slc43a1	AU020206	Resf1	Vim	Marchf3	Prr11	Cacnb4	Gm30211	Ccne2	Nkg7
Sema3d	Rin2	Srgap1	Gm57304	9930111J21Rik2	Ifngr1	Cxcr6	Ttk	Ly6a	Thra	Gtse1	Mad2l1
Lrrc32	Ctsw	Ikzf4	BC053393	Tbc1d2	Ccr5	S100a4	Scpep1	Foxd2os	Sdcbp2	Gmnn	Cit
Prrt1	Dnai4	Gm11707	Cass4	Fdft1	Plxdc1	Lingo4	Cdkn3	Ncapg	Lgals3	Cenpw	Cdc45
Gm20559		Arsg	Rhoj	Saraf	Adam8	Acsbg1	Espl1	Gm20627	Birc5	Eme1	Cenpk
		Actg2	Manf	Gm39114	Ccr2	Pim1	Gm17767	Igkc	Gbp3	Parpbp	Arhgap19
		Pard6g	Sgms1	Mast3	Tnfrsf1b	Gm48236	Ccnb2	Il1rl1	AW112010	Cdca8	Rad54b
		Mtss1	Tmem135	Trib2	Laptm4b	Ankrd55	Siglece	Il1rn	Bspry	Ankle1	Gen1
		Igf2r	Gpr15	Rell1	Lgmn	Nxpe4	Rtn4rl1	Sgip1	Fabp5		
		Capn3	Peak1	Cyb5a	Cep112	Kctd12	Lztfl1	Ehd1	Cryba4		
		Xkrx	Slc4a7	Iqgap2	Prdm1	Fmnl2	Lrba	Rap1gap2	Gm36660		
		Fcmr	Asxl1	Gm38405	Aqp3	Arnt2	Cdc6	Gm57030	Gcnt2		
		Gm33782	Dtx1	Il10ra	Gm38411	Ccr9	Plin2	Pde2a	Mki67		
		Nr1d2	Jakmip1	Ahnak	Adap1	Gm13522	Esco2	Jcad	Kif4		
		Epdr1	Aopep	Bmpr1a	Ablim3	Rora	H2ac24	Anln	Brip1		
		Zfp608	Itm2b	Rnf17			Rexo5	Shcbp1	Crip1		
		Syp	Ppp1r3b	Crtc3			H2ac4	Msrb3	Ctla2b		
		Smad7	Cst3	Vcl			Septin11	Depdc1a	Trp73		
		Rasl11b	Dtnb	Ctla4			Ckb	Mxd1	Nuf2		
		Slc28a2b	Rap1b	Rad51b			Prc1	Stmn1	Gm29684		
		2610204G07Rik	Gm15987	Gm10874			Gm45052	Ern1	Irf8		
		Ankrd6	Diaph1	Padi2			5730420D15Rik	Sgo1	E2f2		
		Gm35363	Dgkd	Fryl			Mis18bp1	Neil3	H2ac15		
		Tspan2	Ubash3b	Galnt2			Gm9888	Hlf	Mastl		
			Kcnk7	Ccs			Slc17a8	Niban2	3300005D01Rik		
			Slc45a4	Pitpnc1			Pola1	Gpr55	N4bp1		
			Dusp5	Esyt1			Fancd2	Atf6	Lgals1		
			Sema4d	Rrad			Ffar2	Tmeff2	Kntc1		
			Dhrs3	Ly6g5b			Racgap1	Irak3	Uhrf1		
			Peli1	Il2rb			Gmfg	Spag5	Nav1		
							E2f7	ENSMUSG00000121481	Slc36a3os		
							Tead1	Hmmr	H1f5		
							Cpm	Cenpp	Cacna1i		
							Ifitm3	Fbxo30	Piwil2		

Supplementary Table 3

DEGs suppressed by TGF-β and IL-2 signaling (padj < 0.0001, $log_2FC > 1$)

TGF-β alone		TGF-β + IL-2						IL-2 alone						
83	49	436					47	428					120	
4933406J09Rik	Cdca3	Aldh5a1	Slc25a13	Iigp1	Grtp1	Gm49692	Cd200	Itm2a	Amacr	Fap	Prkca	Gna14	Chil5	Gm19261
Fam81a	Cdc25c	Gm33195	Prxl2b	Neurl2	Oasl2	Per2	Dgki	Exph5	Ggt5	Bhlhe40	Gm28187	Arhgap24	Gm56767	Ears2
Ntng1	Tpx2	Slc2a6	Samd15	Dlg2	Pcgf5	Efna4	1700019D03Rik	Bcl7a	Cybb	Shisa6	AB124611	Gm13483	Ovgp1	Reep2
Agr3	H2ac11	Arrdc4	Ift22	Afp	Gstm4	Nubpl	Cacna1d	Mdk	Csf2ra	Scmh1	Casp4	ENSMUSG00000121434	A230083N12Rik	Treml2
Gm32081	Cenpe	Gm30214	F13a1	Col24a1	Ddah1	Csgalnact1	Ms4a4c	Tmem38b	Mgmt	Cask	Gm14377	Pam	Gm28068	H2-DMb2
Kirrel3os	Ccl5	Hspa12a	Gm49144	Ak4	Tnfrsf23	Ptpdc1	Ascl2	D830030K20Rik	Zfp821	Cd160	E130102H24Rik	2900089D17Rik	Rsph10b	Stra6
Gm2000	Ckap2l	Kcnq5	Begain	Sfxn4	Bcl2l14	0610040F04Rik	Tnfsf4	Hmgn3	Gm11290	Gm49967	Fhdc1	Parp3	Gata1	Rragd
Angpt1	Prr11	Slc35g1	Nrbp2	Rnf19a	Zfp518b	Rel	Tshz2	Gm42809	Cers6	Tdrp	Lrp12	Atp8b4	5830468F06Rik	Gm20275
Garin3	Cdca5	Mtln	1700052K11Rik	Luzp1	Rogdi	Sesn3	Ralyl	Jade2	Dph1	ENSMUSG00000121393	Ift172	Fgf2	Fndc10	Cd83
Crtam	Gm57152	Gm15918	Prss36	Pde8a	Shf	Prss2	Vat1l	Chrna1os	Morn1	Hectd2	Gm12840	Bank1	1700019L13Rik	Grik4
Slc30a3	Ccnb1	Dusp6	Cd82	Nme3	Ptpn13	Bmp7	Rassf8	Gm49794	Pou2f2	Tgfbr3	Ankrd46	Nfix	Ntn1	Gm28942
Zfhx3	Ankle1	4933406I18Rik	5330426L24Rik	Ldhd	Id1	1810041H14Rik	Gm57070	Tmem158	St8sia6	Ciart	Map7	Tmie	Wls	Rgs9
Gm57169	Bub1	Max	C78197	Zan	Fam228b	Fhit	4930554G24Rik	A630014C17Rik	Rwdd3	Tspan6	Gpr45	4833447P13Rik	Scd4	Susd1
Pak6	Pclaf	Casz1	Gm29477	Gm56689	Egf	Dusp2	Prelid2	Gm32126	Dnal1	Gm33728	Ipo4	Pou6f1	Tnfrsf26	Ikzf2
Thbs1	Spc24	Plscr3	Fam222a	Tgtp1	Klf4	Mccc2	Eomes	H2-Q5	Mapk12	Art3	Ube2e2	Gm57292	Rgl1	Cdc42ep4
Csf1	Nusap1	Cd274	Idh2	Gm31812	Gm50221	Gm20535	Tnp2	Bicdl1	Chst15	Esr1	Prickle1	Ms4a6c	Ksr2	Ldlrad3
Ccser1	Nkain2	Gm16754	Orai1	B3galt1	Gm13375	Ccdc191	Slamf7	Smim10l2a	Pdlim1	Gm47512	Gm48671	Pstpip2	BC065397	Agbl1
Ptgir	Eldr	E030042O20Rik	Gm4792	Lima1	C430002N11Rik	Tnfrsf21	Gm34316	Gm5134	Ccdc28b	Farp2	Gm3696	Prpf40b	Swap70	Tmem38a
Gm35748	Slco4a1	Gm44169	Gm56904	D630008O14Rik	Hes5	Hesx1	Pdzrn3	Hdac9	Gm3752	Acot6	Ptger2	Bcam	Plcl1	Myh15
Ptprn2	Gm42031	C1qtnf4	Mir155hg	Sspo	Gm45155	Gramd1b	Gm2762	Trpm6	Casp1	Gm34093	Zfp773	A230072E10Rik	Sardh	Slc22a2
Capsl	Shcbp1	Rps28	Egr4	Pus7l	Gm56632	D630036H23Rik	Pard3b	Lzts1	Mettl1	Ptpn5	Nhsl1	Dok3	Cmya5	Lhfpl3
Il2	Sgo1	E230032D23Rik	Gm56944	Hopxos	Gm26672	Adssl1	Il4	Zfpm1	Ms4a6d	Zdhhc1	Gm57230	Susd4	Gm56862	Rec8
Sgms1os1	2610318N02Rik	Gm50455	Zfp93	Mmachc	Gm29676	Mtfp1	Shtn1	Synpo	Tbc1d4	Gm13256	Ttc28	Slc35f5	Igfbp4	Gxylt2
Gm50410	Kif4	Elapor1	Top1mt	Plekho1	Nphp1	Ephx4	Sostdc1	Prdm5	Ptpn11	Ica1l	Itga9	Tmem8b	Cc2d2a	Eno3
Akap5	Ifitm2	Gstp3	Marchf8	Gm43434	Gm31108	Sbsn	Hs6st3	Plxdc2	Gm26571	Myo1h	Ddc	9430078K24Rik	Adcy6	1700027J07Rik
Gstm1	Ube2c	Cd22	Meg3	Rab39	Dync2i2	Vmn2r96	Ifi44	Galnt9	App	Pou2af1	Sh3gl3	Pter	Gm48521	Smyd5
Spp1	Lgals7	G0s2	Opn3	Zfp516	Zc3h6	Spo11	Chst3	Sox4	Fam43a	Boll	Gm26759	Klhdc8b	Shb	Spaca6
Sema4c	4930549P19Rik	Tmem88	Gm16150	Gm57038	Coq4	4833419F23Rik	Lpar3	Immp2l	Ccdc112	2610307P16Rik	Gm7967	Gm56560	Fcrl1	Noxa1
Mfsd2a	H3c3	Acad12	Gm56680	Alox12	Zfp428	Kif13a	Adamts20	Wdr35	Mctp1	Ddx43	Igf1r	Ccdc122	Fam174b	Cnga1
Mei1	Tbx21	Gm42067	Lrrc51	Khk	Dync1i1	Ager	Stc2	Arhgap29	Gm41757	Cxxc5	Dlg3	Evl	1110032F04Rik	Palld
Chn1os3	Ccnb2	Btf3l4b	Trav14-1	Alpk1	Gm4668	Morn2	A330093E20Rik	Gm41804	Ctsl	1810006J02Rik	Zfp946	Decr1	H2ac6	Tgm2
Crygn	H2ac24	Gm26826	Arhgef33	Gm13528	Nutf2-ps1	Rps29	Gm46519	Scg5	Erich3	2010007H06Rik	Chrm3	Snx10	Tns1	Kcnip2
Gzmk	H2ac4	Sh3rf1	Flrt2	Gm57201	9230116N13Rik	Dio3os	Gm47957	Ccr7	Nr3c2	Cfap20dc	Pheta1	Ccdc50	Gadd45g	Bicd1
Edn3	Plek	Chchd6	Slc9b1	Gm5106	Gm11342	Fhip1a	C030005K06Rik	Dapl1	Calhm6	Gm57385	Sp110	Gm34343	Cd96	Tmem108
Samd3	H2ac8	Trmt61a	Dync2li1	Il1rl2	1700027F09Rik	Gm56513	Plpp1	Zc3h12b	Btla	Gm45353	Trpm1	Gm49041	Platr29	Magi2
Apoe	Sapcd2	Carmil1	Sh3bp2	Btbd16	Usp6nl	Bcl2a1d	Padi4	Tceal3	Gjb2	Ift122	Cebpa	Gm15411	Gm11999	5430402O13Rik
Tubg2	E2f7	Ptprk	Zfp612	1810019N24Rik	Gm13293	Oaf	Il21	Asap1	Gemin8	Xkr6	Gm9895	Dipk1b	Qtrt2	2010204K13Rik
Nr4a2	Parpbp	Ocln	Gm4577	Cyb5rl	Bend3	Gm36839	Nrn1	Qser1	Gm47861	Gm3667	Trub1	Man1c1	Naglu	Cstpp1
Bend4	Kif15	Apex1	Pkhd1l1	Ccdc40	Eif3j2	Efcab5	Tex15	Rnf122	Aldh6a1	Cryzl2	Gm2237	Dip2c	Zbtb10	Lrig1
Myh10	Ociad2	Arl4d	Thbs3	Bend5	Phb	Bzw2	Pawr	Map9	Six5	H2-Oa	Arhgef9	Ryr2	Capn3	Actg2
Chn1	Ccna2	Rcl1	Iqsec2	Gm26760	Zbed3	Gnl3	Gm50287	Ssbp2	Gm21057	Gm26887	Chn2	Tmem220	H2-DMb1	Efnb1
Art2a	Ska1	Adcy9	Eid2	Ifngas1	Bbs2	Plekha8	Smo	Rpp38	Gm15708	Whrn	Zbed5	Apol7e	Ccdc88a	Myo1e
6430562O15Rik	Rad51ap1	Gm56640	Rian	A930002I21Rik	Fst	Gm15834	Ifng	Tiam1	Ehd3	Cnr2	Gm53065	Nrgn	St8sia1	Slc25a27
Tmprss4	2810408I11Rik	Napb	Gm15747	Syne4	D130017N08Rik	Vps13a	Stfa3	Plp1	Apobec1	Vwa5a	6330403K07Rik	Fam131a	Adamtsl4	Pde5a
Susd2	Hmmr	Mfsd13a	Arhgap32	Rps17	Tspear	Gm49896	Lancl3	Ldhb	5930430L01Rik	Npas4	Gm31718	Gypc	Slc7a10	Adipoq
Trim36	Birc5	Gm56848	Gm41386	Cbarp	Zfp661	2410018L13Rik	Rbpms	Car11	Bphl	Tmem243	Gm13842	Nsg2	Gm56573	Mfsd2b
Gm11867	AI854703	Gm36220	Gm35315	Prx	Gm56824	2310068J16Rik	Aldh2	BC106179	Pank1	Slc25a42	Car7	Sp140	Gm13091	Cyth3
Ifi211	Nkg7	Adamts17	Gm1123	Cryz	Gm28935	Macrod1		A330040F15Rik	Gm49180	C230024C17Rik	Plk2	Tmem17	Ttn	Utf1
Crppa	Ryk	Dach2	Cdkn1c	Gm15133	Pkn3	Mocs3		Pdzk1ip1	Gm56719	Tet1	Gm49179	Pus7	Zfp354c	Gm9889
Gm33460		Gm12184	Spata7	Abhd8	Cfap418	Rnd3		Zfp783	Plscr1	Efnb2	Gm47015	Gm15952	Bmyc	Smpdl3b
Gm4841		Evi5	Chil6	Gm36608	Zfp607b	Chadl		Gm28809	Ccnd3	Lypd6b	A630001O12Rik	Serpinb9	Ift80	Sh3bgrl2
Kcnc2		Olfml3	Usp27x	Cebpd	Rps27	Poglut3		Dock6	Icam5	Gm47941	Ly6m	Gramd3	Ampd3	Cd1d1
Gm51802		Intu	Cnrip1	Ift88	Ap1m2	B3gnt2		Myc	Dzip1	Sh2b3	Gpm6b	Bcl9	Rnf130	Thnsl1
Acy3		Zfp703	Isoc2a	Fam120aos	Ifit1bl2	Sult2b1		Ly6c1	Tmem86a	8030451A03Rik	Gm17455	C130026I21Rik	Rhox8	Zfp608
Ccn4		Rap1gap	Gm57462	Mypop	Gm56772	Snhg9		Arc	Brdt	Klhl3	Gm29114	Prrg4	2610204G07Rik	Patj
Gucy2g		Tenm4	Heatr3	Kyat3	Gm56868	Pax5		Gprc5b	Peak1os	Dnase1l3	Ckap4	4930431P03Rik	B430105G09Rik	Pid1
Akap6		Krt222	Mcrip2	Zcchc12	Gm16152	Spaca7b		Wdr19	Tnfsf11	Ankrd13b	Mctp2	Pim2	Tnfrsf9	Irf2bp2
Wnt3		Tnnt1	Unc93b1	Dhdh	Gm57321	Ttll9		Clip3	Tespa1	1700029J07Rik	Fndc3b	Spry1	Gm46280	P3h1
Kcnn2		Cd40lg	Rabl2	Slc30a4	Gm14440	Bloc1s1		Bcas1	Gstt3	Kcnip1	Zfp976	Hgf	1700025G04Rik	Zfp449
Slc1a4		Gm11655	A230059L01Rik	Fam229b	Gm13563	Ccl27a		Gabrr2	Or5m3b	Gm10634	Gm3739	Dnm3	Gm56901	Bmf
A730063M14Rik		Grwd1	Zfp239	Spin4	Gm31814	Ahi1		Gm56665	Sptb	Lif	Cd9	Fbxo36		
Xdh		Gm56655	Gm56911	Traj11	Ift74	Rpl35		ENSMUSG00000121485	Snn	Ddx60	Lipt2	Gm33619		
Tm6sf1		Gm15860	Agbl3	Egr3	Rab11fip5	Dse		Bcl2	Nphp3	Gm30564	Cfap77	Dync2i1		
Uba52		Fasl	ENSMUSG00000121424	Pxylp1	Pdcd1lg2	Tsacc		Ttc8	Ablim2	Pacsin1	Gab2	Gm11998		
Tsbp1		Pdf	Gm31616	Dnah12	Usp18	Eml6		Dthd1	Gm34006	Gucy1a1	Nbn	Id3		
Gm2464		Smyd2	Nhlrc1	Abcd3	Abhd4	Phldb1		Frmd4a	Hdgfl3	Nod1	Smad1	Gm57398		
Mnd1		Lacc1	Trbj1-2	Dram1	Gm56714	Adamts3		Vipr1	Pdk2	Ppic	Arhgap5	Gm50211		
Ccdc80		Gm10131	Coq8a	C2cd4b	Cstad	Degs2		Ces2c	0610043K17Rik	Dock4	Cpq	2310001H17Rik		
Scg2		Tlcd2	Metap1d	Rps27rt	Tpd52	Tmem150a		Tpmt	Tnfsf8	Spata48	Ttc3	Map2k6		
Gm49708		Gm16675	Hhex	Cdk2ap1	Rgs8	Pigr		Gm15856	Tnfsf14	Gm17173	Ms4a4b	Srm		
F830016B08Rik		4930469K13Rik	Fbn2	Gm56577	Rnf128	Pabpc4		Bcat1	Chpt1	5730471H19Rik	Tcf7	1700041G16Rik		
Ifi204		Gm57227	Tspan31	Gm2296	Gm31135	Gm56698		Pecr	Kirrel3	Car12	Nr4a1	Gm14718		
Klrb1a		Gm16230	Dkc1	Ppfibp2	Htr2a	Lekr1		B4galnt1	Gm57351	Marcksl1	Ptk2	Phpt1		
Ccdc141		Gm57078	Ago4	C530005A16Rik	Atg4c	Mapk11		Tfap4	Gm16120	Prss41	Gm36459	Qrfp		
Il12rb2		Ctxn1	Polr1g	Ptrh1	Trdj1	Ifih1		Parp8	Gm17066	Gsta4	Tnfsf13b	Gm33936		
Hectd2os		Gstk1	Sox5	Zfp651	Celsr1	Rpl10-ps3		Tgif2	Cldn10	Plagl1	Slc16a1	Maged2		
Pmvk		Gm43581	Epop	Cd109	Insl6	Gsap		Scamp1	1300014J16Rik	Gm56842	Gm14636	Slc29a1		
Spire1		Lad1	Pla2g4f	Snhg6	9930014A18Rik	Nfkbiz		Dennd2d	Txnrd3	Amigo2	Aff3	Sugct		
A530010L16Rik		Sidt1	Gm3264	Ppan	Zcwpw1	Thop1		Rgmb	Zfp975	Dnajc12	Bdh2	Cetn4		
1700011L03Rik		Pebp1	Gm42620	Gm15283	Gm50237	Thrb		Mboat1	Cnn3	Hecw2	Tnc	Gm56730		
Enpp2		4932412D23Rik	Smim27	Asnsd1	Gm31504	Ticam2		Arhgef5	Egr2	Afap1	Arhgap20	Dctd		
Gm43057		Arfgef3	2610042L04Rik	Ptges3l	Oas1a	Kif9		Erf	Tmcc3	Rnaset2a	Rida	Ssh3		
Gm12534		H2-Q10	1110028F11Rik	Nefh	Gm57347	Ptov1		B630019A10Rik	H2-Q6	Ier5l	Pagr1a	Slc9b2		
		Il9r	Fhad1	Phlda3	Gm16759	Zfp820		Rangrf	Gucy1b1	Armc3	Lhpp	Adk		
		Tjp2	1700019A02Rik	Ndufb4	Dglucy	Sfxn5		Gm35188	Gm56797	Ccdc148	Apobec2	Gm44148		
		Akr1c12	Bcl6	Gm34150	Ier3	H2-Q7		Zfp974	Scml4	Penk				
		Sh2b2	Gm56740	2010016I18Rik	2900009J06Rik	Gm10406								
		Cul9												
