# Supplementary material for: Facile induction of immune tolerance by an interleukin-2–TGFβ surrogate agonist
Source: Nature. 2026 Mar 11;653(8115):888–99. doi: 10.1038/s41586-026-10208-0 (PMC13190267; doi:10.1038/s41586-026-10208-0)
Supplement: Supplementary file 2 — Reporting Summary [file 41586_2026_10208_MOESM2_ESM.pdf]

nature portfolio

Corresponding author(s): K. Christopher Garcia

Last updated by author(s): Jan 12, 2026

Reporting Summary

Nature Portfolio wishes to improve the reproducibility of the work that we publish. This form provides structure for consistency and transparency in reporting. For further information on Nature Portfolio policies, see our Editorial Policies and the Editorial Policy Checklist.

Statistics

For all statistical analyses, confirm that the following items are present in the figure legend, table legend, main text, or Methods section.

n/a	Confirmed	
☐	☒	The exact sample size (n) for each experimental group/condition, given as a discrete number and unit of measurement
☐	☒	A statement on whether measurements were taken from distinct samples or whether the same sample was measured repeatedly
☐	☒	The statistical test(s) used AND whether they are one- or two-sided<br>*Only common tests should be described solely by name; describe more complex techniques in the Methods section.*
☒	☐	A description of all covariates tested
☐	☒	A description of any assumptions or corrections, such as tests of normality and adjustment for multiple comparisons
☐	☒	A full description of the statistical parameters including central tendency (e.g. means) or other basic estimates (e.g. regression coefficient) AND variation (e.g. standard deviation) or associated estimates of uncertainty (e.g. confidence intervals)
☐	☒	For null hypothesis testing, the test statistic (e.g. F, t, r) with confidence intervals, effect sizes, degrees of freedom and P value noted<br>*Give P values as exact values whenever suitable.*
☒	☐	For Bayesian analysis, information on the choice of priors and Markov chain Monte Carlo settings
☒	☐	For hierarchical and complex designs, identification of the appropriate level for tests and full reporting of outcomes
☒	☐	Estimates of effect sizes (e.g. Cohen's d, Pearson's r), indicating how they were calculated

Our web collection on statistics for biologists contains articles on many of the points above.

Software and code

Policy information about availability of computer code

Data collection	Flow cytometry data were acquired using a CytoFLEX (Beckman Coulter). scRNA-seq libraries were sequenced on an Illumina NovaSeq X. Tissue sections were imaged using a Leica DM2000 microscope.
Data analysis	The FASTQ files were processed using Cell Ranger v9.0.0. The gene expression matrix was processed and analyzed using Seurat (version 5.1.0). For quality control, we excluded cells that contained fewer than 500 read counts for genes or fewer than 200 genes detected (minimal cutoff), or more than 50,000 read counts for genes or more than 6500 genes detected (maximum cutoff). We also excluded cells in which more than 20% of transcripts were derived from mitochondrial RNA. These QC filters left 190,728 cells. Graph-based unsupervised clustering was employed to identify clusters representing minor contaminant cells other than T cells, such as neurons (expressing Cntn1, Dscam, and Pde7b) and B cells (expressing Igkc, Ms4a1, and Cd79a). These minor clusters were excluded from subsequent analyses, leaving 180,038 cells. Uniform Manifold Approximation and Projection (UMAP) embedding was computed with 10 principal components, with n.neighbors being 20 and min.dist being 0.1. Differential expression (DE) analysis between groups was performed using Wilcoxon's rank sum test implemented in Seurat's FindMarkers function. Gene set enrichment analysis (GSEA) was performed using the log2FC ranking of DE genes using fgsea. Human Hallmark gene sets were retrieved from MSigDB. The signature scores were calculated using Seurat's AddModuleScore function. To calculate Cell Cycle score, Seurat's CellCycleScoring function was used. Built-in human genesets for the S and G2M phases in Seurat were converted into mouse homologues and used to calculate S and G2M scores. Transcription factor activity inference was conducted using pySCENIC with default parameter settings. To identify DEGs from public bulk RNA-seq datasets for computing gene-set signature scores, bulk RNA-seq FASTQ files were aligned to the GENCODE VM25 (mm10) reference genome using Rsubread, and gene expression was quantified with featureCounts. DE analysis was performed using DESeq2. Pathway analysis was performed using Metascape. The R code used to analyze the scRNA-seq data has been deposited on Zenodo (10.5281/zenodo.18166788). Analysis details are provided in the Methods.

For manuscripts utilizing custom algorithms or software that are central to the research but not yet described in published literature, software must be made available to editors and reviewers. We strongly encourage code deposition in a community repository (e.g. GitHub). See the Nature Portfolio guidelines for submitting code & software for further information.

Data

Policy information about availability of data

All manuscripts must include a data availability statement. This statement should provide the following information, where applicable:

- Accession codes, unique identifiers, or web links for publicly available datasets
- A description of any restrictions on data availability
- For clinical datasets or third party data, please ensure that the statement adheres to our policy

The raw and processed scRNA-seq data have been deposited in the Gene Expression Omnibus (GEO) under accession GSE315102 (aligned to the mm10 mouse reference genome). The R code used to analyze the scRNA-seq data has been deposited on Zenodo (10.5281/zenodo.18166788). Analysis details are provided in the Methods.

Research involving human participants, their data, or biological material

Policy information about studies with human participants or human data. See also policy information about sex, gender (identity/presentation), and sexual orientation and race, ethnicity and racism.

Reporting on sex and gender	Gender information was not collected.
Reporting on race, ethnicity, or other socially relevant groupings	N/A
Population characteristics	Human T cells were isolated from buffy coats obtained from anonymous healthy donors (male and female; sex was not recorded) purchased from the Stanford Blood Center.
Recruitment	Anonymous healthy donors were recruited by the Stanford Blood Center.
Ethics oversight	Ethical approval pertaining to donors was obtained by the Stanford Blood Center.

Note that full information on the approval of the study protocol must also be provided in the manuscript.

Field-specific reporting

Please select the one below that is the best fit for your research. If you are not sure, read the appropriate sections before making your selection.

☒ Life sciences ☐ Behavioural & social sciences ☐ Ecological, evolutionary & environmental sciences

For a reference copy of the document with all sections, see nature.com/documents/nr-reporting-summary-flat.pdf

Life sciences study design

All studies must disclose on these points even when the disclosure is negative.

Sample size	Group sizes for in vivo and in vitro validation experiments were determined based on prior knowledge of expected variability, with a minimum of three mice per group.
Data exclusions	Rout outlier tests were run with default parameters (Q = 1%) in Prism on all mouse experimental data due to inherent variability within the model system.
Replication	All presented results were repeatable. Replicates were used in all experiments as noted in figure captions or methods.
Randomization	Age and sex-matched animals were used for each experiment. Mice were randomized prior to treatment. In the in vitro experiments, samples with same pretreatment conditions were randomly assigned to a treatment group.
Blinding	Histological analyses were performed in a fully blinded manner. Blinding was not performed for the remaining experiments due to requirements for cage labeling and staffing constraints.

Reporting for specific materials, systems and methods

We require information from authors about some types of materials, experimental systems and methods used in many studies. Here, indicate whether each material, system or method listed is relevant to your study. If you are not sure if a list item applies to your research, read the appropriate section before selecting a response.

Materials & experimental systems

n/a	Involved in the study
☐	☒ Antibodies
☐	☒ Eukaryotic cell lines
☒	☐ Palaeontology and archaeology
☐	☒ Animals and other organisms
☒	☐ Clinical data
☒	☐ Dual use research of concern
☒	☐ Plants

Methods

n/a	Involved in the study
☒	☐ ChIP-seq
☐	☒ Flow cytometry
☒	☐ MRI-based neuroimaging

Antibodies

Antibodies used

The following antibodies were purchased from BioLegend: mouse CD45.2 (109839), CD3 (100206), CD4 (100453, 100430, 100428, 100451), CD8 (100706), NK1.1 (156506), TCRvα2 (127806, 127822), TCRvα3.2 (135404), Thy1.1 (202528, 202522), CTLA4 (106310), CD62L (104453), CD25 (102012, 102022, 102047, 102038), CD44 (103026, 103032), SIGLECF (155534), CD73 (127215), ICOS (313550), CD69 (104530), CD11b (101259), CXCR3 (126514), CD39 (143806), NRP1 (145218), CD11c (117318), CD103 (110910), GITR (126316), CXCR6 (151117), CCR6 (129819), IL-17A (506928), GMCSF (505406), IFNγ (505832, 505826), IL-10 (505034, 505026, 505034), BLIMP1 (150008), Helios (137214), Ki-67 (151212, 652406), TNFα (506346); human CD3 (317324), CD4 (980806) and FOXP3 (320126). The following antibodies were purchased from BD Biosciences: mouse BCL6 (562401), RORγt (564722, 562682, 562683), SMAD2 (pS465/pS467)/SMAD3 (pS423/pS425) (562696) and STAT5 (pY694) (612599). The following antibodies and reagents were purchased from Invitrogen: mouse PD-1 (48-9985-82), FOXP3 (12-5773-82, 17-5773-82, 404-5773-82), T-bet (25-5825-82), c-MAF (53-9855-82), GATA3 (46-9966-42) and Fixable Viability Dye (65-0865-18). PE- and Brilliant Violet 421-labeled I-Ab OVA328-337 tetramers (HAAHAEINEA) were provided by the NIH Tetramer Core Facility.

Validation

All commercially available antibodies were validated by the manufacturers.

Eukaryotic cell lines

Policy information about cell lines and Sex and Gender in Research

Cell line source(s)

The Expi293F™ cells were purchased from Thermo Fisher Scientific.

Authentication

None of the cell lines were authenticated in these studies. In all studies, cell lines with low passage number were used.

Mycoplasma contamination

All cell lines were confirmed mycoplasma negative.

Commonly misidentified lines
(See ICLAC register)

No commonly misidentified cell lines were used.

Animals and other research organisms

Policy information about studies involving animals; ARRIVE guidelines recommended for reporting animal research, and Sex and Gender in Research

Laboratory animals

Six- to eight-week-old female and male C57BL/6J mice (IMSR_JAX:000664), as well as other strains, were purchased from The Jackson Laboratory. OT-II (IMSR_JAX:004194) and Thy1.1 (IMSR_JAX:000406) mice were crossed to generate OT-II Thy1.1 mice. Foxp3-GFP mice (IMSR_JAX:006772) were crossed with OT-II Thy1.1 mice. 2D2 mice (IMSR_JAX:006912) were crossed with Thy1.1 mice. All animals were housed in AAALAC-accredited facilities.

Wild animals

No wild animals were involved.

Reporting on sex

Female mice were used for EAE experiments. In all other experiments, both male and female mice were used, and no sex-specific phenotypes were observed.

Field-collected samples

There were no field-collected samples.

Ethics oversight

All experimental mouse procedures were approved by the Stanford University Institutional Animal Care and Use Committee (IACUC; protocol IDs 32279 and 34708) and conducted in accordance with institutional guidelines.

Note that full information on the approval of the study protocol must also be provided in the manuscript.

Plants

Seed stocks: N/A

Novel plant genotypes: N/A

Authentication: N/A

Flow Cytometry

Plots

Confirm that:

☒ The axis labels state the marker and fluorochrome used (e.g. CD4-FITC).

☒ The axis scales are clearly visible. Include numbers along axes only for bottom left plot of group (a 'group' is an analysis of identical markers).

☒ All plots are contour plots with outliers or pseudocolor plots.

☒ A numerical value for number of cells or percentage (with statistics) is provided.

Methodology

Sample preparation: Mouse lymph nodes and spleens were harvested and mechanically dissociated to obtain single-cell suspensions. Red blood cells were lysed using ACK lysis buffer (A10492-01, Gibco), followed by magnetic isolation of $CD4^+$ T cells using the EasySep™ Mouse $CD4^+$ T Cell Isolation Kit (19852, STEMCELL). Naïve $CD4^+$ T cells ($CD4^+CD44^-CD25^-$Foxp3-GFP^-) and activated $CD4^+$ Tconv cells ($CD4^+CD44^+CD25^-$Foxp3-GFP^-) were subsequently sorted using a Sony SH800S Cell Sorter. The purity of the sorted populations was consistently greater than 99%. Human $CD4^+$ T cells were isolated from frozen PBMCs using the EasySep™ Human $CD4^+$ T Cell Isolation Kit (17952, STEMCELL). BALF was collected by flushing the lungs three times with 0.75mL of PBS via a catheter inserted into the trachea. For lymphocyte isolation from the lung, tissues were mechanically dissociated using the plunger of a 1mL syringe and filtered through 70µm cell strainers to obtain single-cell suspensions. For lymphocyte isolation from the lamina propria, Peyer's patches in the small and large intestines were first removed. The intestines were then opened longitudinally, cut into ~2-cm pieces, and incubated in 5 mM EDTA (15575020, Invitrogen) with 1 mM DTT (R0861, Thermo Fisher Scientific) at 37 °C for 30 minutes to remove epithelial cells. Tissues were then minced and digested in DNase I (40µg/mL; Roche) and collagenase D (0.5mg/mL; Roche) at 37°C for 30min with shaking to generate single-cell suspensions, which were filtered through 70µm cell strainers. For lymphocyte isolation from the spinal cord, mice were first perfused, and the collected spinal cords were mechanically dissociated using the plunger of a 1mL syringe and passed through 70µm cell strainers to obtain single-cell suspensions. The resulting cells were subjected to density gradient centrifugation using a 40%/70% Percoll (Cytiva) gradient. Immune cells located at the interface between the two Percoll layers were harvested and processed for flow cytometry analysis.

Instrument: CytoFlex (Beckman Coulter)

Software: FlowJo (v 10.10.0)

Cell population abundance: Sort purity was determined to be 95% by analyzing a post-sort sample.

Gating strategy: Donor OT-II cells were identified as TCR $V\alpha2^+$ Thy1.1^+, and donor 2D2 cells as TCR $V\alpha3.2^+$ Thy1.1^+. Eosinophils were gated as $CD45^+$ $CD11b^+$ $CD11c^-$ Siglec-F^+. Naive $CD4^+$ T cells were gated from $CD4^+$ $CD44^-$ $CD25^-$ Foxp3-GFP^- cells, and activated $CD4^+$ conventional T cells (Tconv) were gated from $CD4^+$ $CD44^+$ $CD25^-$ Foxp3-GFP^- cells.

☒ Tick this box to confirm that a figure exemplifying the gating strategy is provided in the Supplementary Information.
